# High-dose romosozumab promoted bone regeneration of critical-size ulnar defect filled with demineralized bone matrix in nonhuman primates

**DOI:** 10.1016/j.jot.2025.06.019

**Published:** 2025-07-10

**Authors:** Xiaodong Li, Frank Asuncion, Michael Ominsky, Qing-Tian Niu, Kristina E. Akesson, Jeffrey Wang, Jay Lieberman, Hua Zhu Ke

**Affiliations:** a Amgen Inc., Thousand Oaks, CA, USA; b Orthopedics, Department of Clinical Sciences, Lund University, Malmö, Sweden; c Department of Orthopaedics, Skåne University Hospital, Malmö, Sweden; d University of Southern California, Los Angeles, CA, USA

**Keywords:** Bone regeneration, Critical-size bone defect, Demineralized bone matrix, Nonhuman primates, Romosozumab

## Abstract

**Background:**

Large bone defects are challenging to manage clinically and usually require treatment with bone graft or bone graft substitute. This study evaluated the effect of romosozumab, a sclerostin antibody, in combination with demineralized bone matrix (DBM) on bone regeneration in a critical-size ulnar defect model in nonhuman primates.

**Methods:**

In cynomolgus monkeys (N = 22, male, 10–12 years old), a full-cortex bone defect (0.5 cm long) was created in the left ulnar shaft and filled with DBM. Animals were randomized to receive vehicle (n = 10) or romosozumab (n = 12; 30 mg/kg) subcutaneously, every 2 weeks for 28 weeks. Radiographs of the left ulna were taken every 2 weeks for 28 weeks to monitor bone regeneration response. Ulnae were excised and analyzed by ex-vivo x-ray and micro-computed tomography (micro-CT) to evaluate bone repair, and lumbar vertebrae were excised for bone histomorphometric analysis to evaluate the systemic anabolic response.

**Results:**

In-vivo and ex-vivo x-ray images of surgical ulnae demonstrated that the critical-size ulnar defect fully bridged in 3 romosozumab-treated monkeys at week 28 but not in any vehicle-treated monkey. Micro-CT analysis demonstrated that average new bone volume and new bone area within the defect region were 118 % and 105 % greater, respectively, with romosozumab versus vehicle. Trabecular bone volume per tissue volume and trabecular thickness of lumbar vertebral body were 72 % and 92 % greater, and eroded surface was significantly lower with romosozumab versus vehicle.

**Conclusion:**

High-dose romosozumab in combination with DBM improved bone regeneration in a critical-size ulnar defect model and increased bone mass in non-surgical bone in nonhuman primates.

**The translational potential of this article:**

Clinical management of large bone defect is complex and challenging. More effective management is needed. This paper reports the first nonhuman primate study that evaluated high-dose romosozumab in combination with demineralized bone matrix in a critical-size defect model and provides perspective for the future research evaluating the combination of romosozumab and bone graft or bone graft substitutes in various relevant clinical conditions.

## 1 Introduction

Bone defects are not unusual and most commonly caused by trauma, whereas they also are associated with bone resection due to tumor or infection, and their management is challenging [1]. Treatment can be further complicated in clinically challenging conditions, such as critical-size bone defects, that are too large to heal and often require the use of autologous bone graft [1,2]. However, there are several drawbacks of autologous bone grafting, including increased risk of injury and infection or limited availability. Other treatment options that promote bone regeneration are currently limited [3].

Previous studies using a femoral defect model in rodents demonstrated that the sclerostin antibody (Scl-Ab) increased bone formation and led to complete bridging of critical-size femoral bone defects in a small subset of rats [4,5]. Despite the fact that Scl-Ab increased bone formation and new bone volume within the defect region, these studies suggested that Scl-Ab is not an osteoinductive agent like bone morphometric protein 2 (BMP2). In these studies, the femoral defect was left empty and was not filled with a bone graft or bone graft substitute. Therefore, it remains unclear whether Scl-Ab in combination with a bone graft or bone graft substitute would have had any effect on bone regeneration.

In 2019, the Scl-Ab romosozumab-aqqg (romosozumab) was approved for the treatment of postmenopausal women with osteoporosis at high risk for fracture by the US Food and Drug Administration (FDA). Romosozumab is a bone-forming agent that exerts a dual action on bone by increasing bone formation and decreasing bone resorption [6,7]. In a placebo-controlled trial, romosozumab reduced fracture risk and increased bone mineral density (BMD) in postmenopausal women with osteoporosis [8]. In active-controlled trials, it reduced fracture risk with higher efficacy compared with alendronate [9], and increased BMD and estimated hip bone strength compared with teriparatide in postmenopausal women with osteoporosis at high risk of fracture [10]. However, in randomized controlled trials, romosozumab, similar to other bone-forming agents [11,12], failed to show accelerated radiographic healing of hip and tibial diaphyseal fractures [13,14]. Taken together, these results suggest that bone-forming agents alone may not significantly accelerate the normal fracture healing process in patients.

It remains unknown whether an Scl-Ab could potentially promote bone regeneration in challenging-to-heal conditions. In this study, we evaluated the effect of high-dose romosozumab in combination with demineralized bone matrix (DBM), an osteoconductive bone graft substitute [15], on bone regeneration response in a critical-size long bone defect model in nonhuman primates, a species whose bone remodeling process closely resembles to that in humans.

## 2 Materials and methods

### 2.1 Animals and experimental design

All in-life procedures of this preclinical study were conducted at Guangxi Weimei Bio-tech Co. (Guangxi, China). The study protocol was approved by the Institutional Animal Care and Use Committee, and the study was conducted according to the study protocol and current Guangxi Weimei Bio-tech Co. guidance documents and standard operating procedures.

**Fig. 1:**
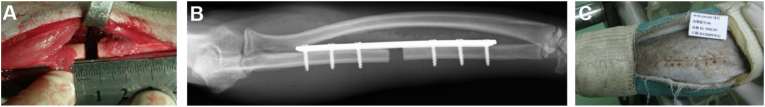
Critical-size ulnar defect A. Representative photograph showing a full-cortex bone defect (0.5 cm in length) created in the shaft of ulna. B. Representative radiographic image of forearm showing the defect of ulna stabilized with a titanium plate and three screws on each side of the defect. C. Postsurgical photograph of fiberglass cast on the forearm.

In cynomolgus monkeys (n = 22; male; 10–12 years old, 6–10 kg), a 0.5 cm long full-cortex bone defect was created in the midshaft of the left ulna that was stabilized with a titanium plate and 6 screws (3 screws on each side of the defect) (Fig. 1). The midshaft of left ulna was chosen since it is known that the midshaft of long bone heals slowly. The bone defect was filled with a fixed amount (8 mm × 1 cm x 1 cm) of DBM (Grafton), and the left forelimb was immobilized with a fiberglass cast in all monkeys. Surgeries were performed under general anesthesia by a qualified orthopedic surgeon and his team according to a standard operation protocol. Each animal was administrated with an intramuscular (im) injection of atropine (0.01 mg/kg) followed by ketamine (3 mg/kg). Anesthesia was maintained using isoflurane and oxygen (O_2_ 3–4 L/min, 4 % isoflurane) through tracheal intubation. All animals were administered buprenorphine (0.015 mg/kg, im) before surgery. An external heating device (e.g. warm air cushion) was used during the entire period of anesthesia and recovery.

**Fig. 2:**
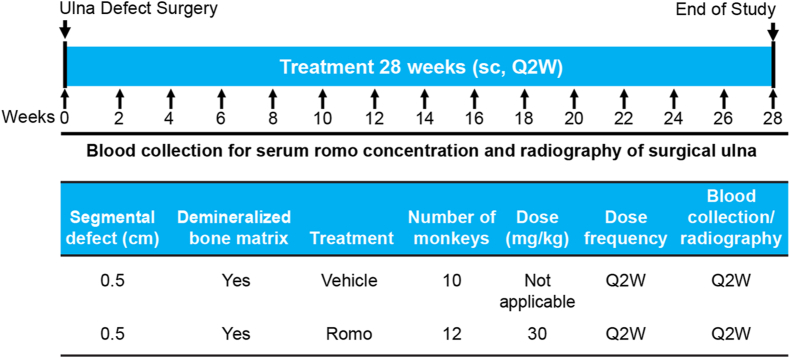
Study design Q2W, once every 2 weeks; Romo, romosozumab sc, subcutaneous.

After surgery, monkeys were randomized into two groups to receive vehicle (n = 10) or romosozumab (n = 12; 30 mg/kg) subcutaneously, once every 2 weeks for 28 weeks (Fig. 2). The dose and frequency were selected to overcome the potential immune response to romosozumab which was based on a previous fracture healing study with romosozumab in cynomolgus monkeys [16]. In this study, romosozumab was administered every 2 weeks at 30 mg/kg. In the bone quality and toxicology studies, romosozumab was administered once weekly at 30 mg/kg in non-human primates for 12 months and at the dose up to 100 mg/kg up to 6 months in the toxicology study, respectively [17,18].

Blood collection to measure romosozumab concentration in the serum and radiography of the surgical ulna was performed once every 2 weeks for 28 weeks, and week 28 was the end of the study (Fig. 2). All animals received an additional dose of buprenorphine (0.015 mg/kg, im) at the end of surgery and post-operatively at about 8–12 h apart for 3 days. Additional buprenorphine (0.015 mg/kg) was administered if animals showed signs of pain or discomfort. Antibiotic injection of cefotaxime (50 mg/kg) was administered intravenously before and at the end of surgery and once a day thereafter for 3 postoperative days. Animals were monitored twice daily for 7 postoperative days for pain, discomfort, limited use of limbs, vital signs, inflammation of surgical site.

### 2.2 Animal care

Monkeys were maintained in Association for Assessment and Accreditation of Laboratory Animal Care internationally accredited facilities and were cared for in accordance with the Guide for the Care and Use of Laboratory Animals as reported earlier [19].

### 2.3 Serum romosozumab concentration

Serum romosozumab concentrations were measured using a bioanalytical method developed at Amgen Inc., Thousand Oaks, CA. Romosozumab in study samples was captured by immobilized biotinylated recombinant human sclerostin. Horseradish peroxidase-labeled recombinant human sclerostin was used to detect captured romosozumab. Tetramethylbenzidine-peroxidase and hydrogen peroxide in the presence of horseradish peroxidase produced a colorimetric signal that was proportional to the concentration of romosozumab.

### 2.4 Evaluation of critical-size defect healing

Radiographs of the left ulna were taken immediately after surgery and every two weeks until the end of the study (28 weeks) (Fig. 2). At the end of the study, the plate and screws were removed from the excised ulnae for ex vivo radiographs (Faxitron) and micro-computed tomography (micro-CT) scanning (GE eXplore Locus SP Specimen Scanner; GE Healthcare). For micro-CT, scans at the midshaft ulna were reconstructed to a 28 μm isotropic voxel size, with contours drawn to span the gap from the ends of the original cortex. Bone volume was determined using the micro-CT software's automatic thresholding algorithm (322–511 mg/cm^3^), with bone area calculated as bone volume divided by defect length. Volumetric bone mineral content (vBMC) was determined without thresholding**.** To monitor bone regeneration response qualitatively, 6-point bridging zoom through analysis was performed on the front and side view of 3D micro-CT images.

### 2.5 Static histomorphometry of the lumbar vertebral body

To confirm the expected effects of romosozumab on non-surgical bone, L2 lumbar vertebrae were excised and histomorphometric analyses were performed as previously described [19]. Static histomorphometric data of trabecular analysis were included in this study, while dynamic histomorphometric data of trabecular and cortical bone have been reported previously [19].

### 2.6 Statistical analyses

All data were expressed as mean ± standard error of the mean. Group differences were determined using unpaired two-tailed Student *t*-test within GraphPad Prism (version 5.01, GraphPad Software, Inc.; San Diego, CA), with statistical significance determined as p < 0.05.

## 3 Results

### 3.1 General health and animal exclusions

Animals tolerated the surgery and antibody treatment well. Serum romosozumab concentrations were above 60 μg/mL from weeks 2–28 in monkeys treated with romosozumab, except for one animal. This animal was excluded for the analysis due to undetectable concentrations of romosozumab from week 6 through the end of the study, likely due to anti-drug antibody–mediated clearance. Serum romosozumab was undetectable in vehicle-treated monkeys at all time points examined.

### 3.2 Radiographic evaluation of critical-size defect

**Fig. 3:**
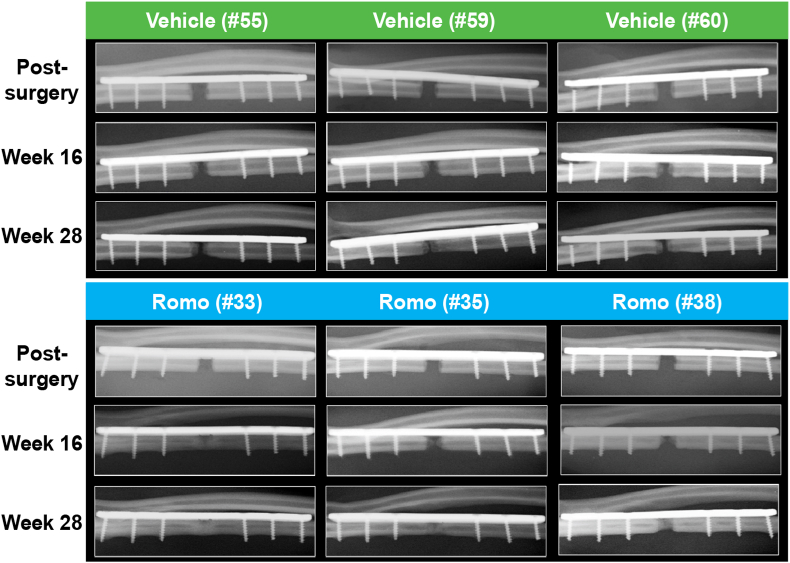
In vivo x-ray images of ulnae with best bone regeneration responses in vehicle- and romosozumab-treated cynomolgus monkeys X-ray images from monkeys treated with vehicle (top) or romo (bottom) post-surgery and after 16 and 28 weeks of drug administration. # = number in the parentheses represents the individual monkey number. Romo, romosozumab.

Fig. 3 and Fig. S1 show the in vivo x-ray images of the 3 best responders in each group over time from weeks 0–28 at selective time points and more detailed time points, respectively. Evidence of bone regeneration appeared to be visible in 2 romosozumab-treated monkeys but was not observed in any of the vehicle-treated monkeys at week 8 (Fig. S1). At the end of the study, at week 28, the critical-size ulnar defect did not fully bridge in any vehicle-treated monkeys; in contrast, the critical-size ulnar defect fully bridged in 3 romosozumab-treated monkeys (Fig. 3 and Fig. S1). Fully-bridged defect represents a condition that there was no visible gap within the critical-size defect region. Fig. S2 shows the in vivo x-ray images of all animals at week 28 in the study. In the romosozumab group, among the 8 monkeys who did not have complete bridging, 5 had visible bone growth within the defect region at week 28 as shown by in vivo radiography (Fig. S2). In contrast, only 2 of the 7 vehicle-treated monkeys (excluding the three best responders) demonstrated visible bone growth within the defect region at the same time point (Fig. S2). Ex vivo radiography supported these in vivo observations, demonstrating greater bridging and bone growth in the defects in the romosozumab versus the vehicle group (Fig. S3).

### 3.3 Micro-CT imaging of the defect region

**Fig. 4:**
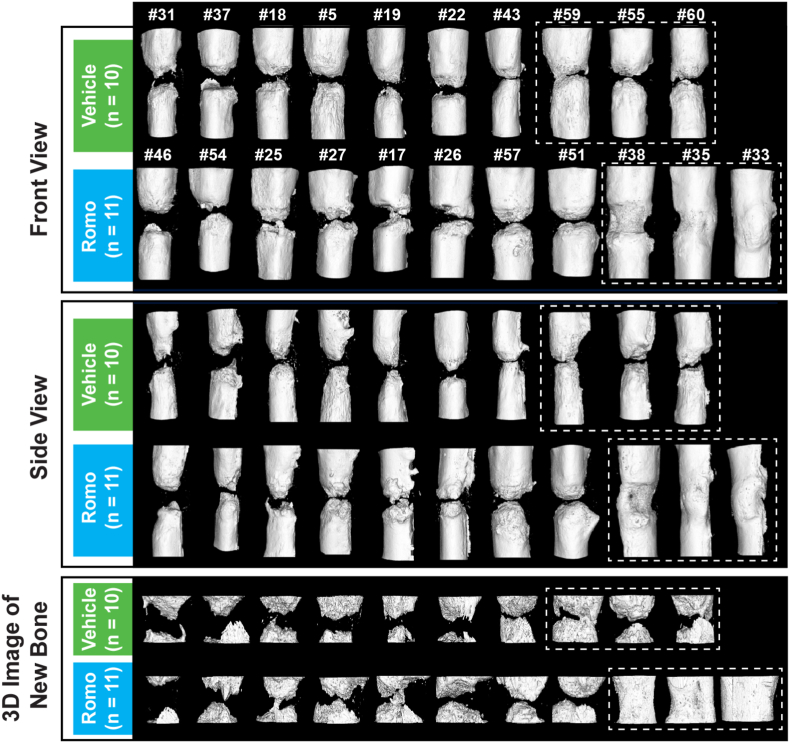
Micro-CT images of critical-size defect regions from all cynomolgus monkeys at week 28 after removal of fixation hardware Images are ranked by values of the new bone area within the defect region from low (left) to high (right). # = number above images represents the individual monkey number. The last three images (inside the boxes) in each group are the best three responders (corresponding to images shown in Fig. 3). One monkey was excluded from the romosozumab group due to undetectable serum romosozumab concentration from week 6 to the end of the study. Romo, romosozumab.

**Fig. 5:**
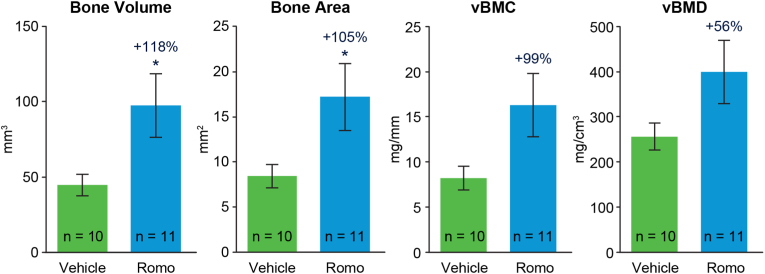
Effect of romosozumab treatment on critical size ulnar defect. Data are represented as mean ± standard error of the mean. ∗p < 0.05 (unpaired two-tailed Student *t*-test). One monkey was excluded from the romosozumab group due to undetectable serum romosozumab concentration from week 6 to the end of the study. Romo, romosozumab; vBMC, volumetric bone mineral content.

Micro-CT evaluation confirmed the findings from radiographic evaluation in the romosozumab group. Micro-CT images of ulna in each individual monkey were shown in Fig. 4 and Fig. S4. Fig. 4 shows front and side view of 3D micro-CT images of ulna and 3D reconstruction of new bone of critical-size defect. Bone defect appeared to be partially bridged in one of the vehicle-treated monkeys, but bridging was observed only in about one-third of the cortical bone (Fig. 4 and Fig. S4). Micro-CT analysis demonstrated that mean new bone volume and new bone area within the defect region were 118 % and 105 % greater in the romosozumab versus the vehicle group, respectively (p < 0.05 for both; Fig. 5). vBMC and vBMD were numerically but not significantly higher in the romosozumab versus the vehicle group (Fig. 5).

### 3.4 Static histomorphometry of the lumbar vertebral body

**Fig. 6:**
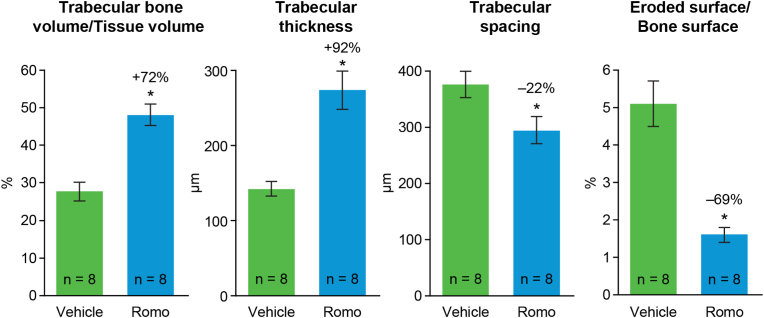
Effect of romosozumab treatment on lumbar vertebral body Data are represented as mean ± standard error of the mean. Romo, romosozumab. ∗p < 0.05 (unpaired two-tailed Student *t*-test). Romo, romosozumab.

Bone histomorphometric analyses of the lumbar vertebral body were conducted in 8 monkeys from each group and showed that trabecular bone volume per tissue volume and trabecular thickness were 72 % and 92 % greater in the romosozumab versus vehicle group, respectively (p < 0.05 for both, Fig. 6). Trabecular spacing was significantly lower (−22 %; p < 0.05) in the romosozumab versus vehicle group. Trabecular number was unchanged (data not shown). Eroded surface per bone surface, an index of bone resorption, was significantly lower (−69 %; p < 0.05) in the romosozumab versus vehicle group.

## 4 Discussion

This study demonstrated that romosozumab in combination with DBM improved bone regeneration in a critical-size ulnar defect in a nonhuman primate model. This study is unique in several aspects. First, this is the first study where romosozumab was evaluated in combination with DBM in a challenging-to-heal condition in nonhuman primates. The size of the ulnar defect and use of osteoconductive DBM simulated a challenging-to-heal condition. Second, the study evaluated the effect of romosozumab in a critical-size defect in a skeletal site that has less weight bearing. Third, the use of rigid internal plate/screw and external fiberglass cast fixations led to healing that was primarily driven by intramembranous ossification, an important mechanism of post-surgical long bone fracture healing in humans [20].

The present study is an important addition to the existing literature on effects of romosozumab on bone regeneration in animal models of critical-size defect. A couple of studies were performed in the rodent models of critical-size defect, but rodents are bone modeling species, and don't have the Harversian system. However, unlike rodents, nonhuman primates are bone remodeling species and have the Harversian system. Moreover, their bone metabolism is closer to human bone metabolism. In addition, the current study is the first one that evaluated the combination of romosozumab with DBM in nonhuman primates. Critical-size defect is very challenging to manage in clinical practice and often requires the use of autograft or bone graft substitute. BMP-2 received approval for clinical use in certain spine fusion procedures in treating certain acute, open tibial shaft fractures and in certain oral-maxillofacial procedures. However, BMP-2 use is very limited in repair of critical-size defect primarily due to amount of BMP-2 required and associated cost. These highlights the continued research need in this area.

In this study, the radiographs of the forelimb demonstrated that none of the vehicle-treated monkeys had a fully bridged defect at the end of the study, while the defect fully bridged in 3 monkeys (25 %) treated with romosozumab in combination with DBM. Micro-CT analysis showed that the new bone volume (+118 %) and new bone area (+105 %) more than doubled in the defect region of romosozumab-treated monkeys compared with that of vehicle-treated monkeys. These results support the concept of evaluating romosozumab in combination with a bone graft or an osteoinductive bone substitute in a challenging-to-heal orthopedic condition. A previous study in rodents appears to support this concept, where the combination of Scl-Ab and recombinant human bone morphogenetic protein-2 (rhBMP-2) resulted in complete bridging of the critical-sized femoral defect in all rats [21]. Animals treated with the combination of Scl-Ab and rhBMP-2 had greater torsional strength and rigidity compared with the unoperated control femur. Our findings appear to be in line with couple recent case reports in human subjects that reported bone union with romosozumab alone or in combination with a bone graft substitute in the management of challenging-to-heal conditions such as delayed union or nonunion [22,23]; however, further studies are required to substantiate these results. Cellular mechanism that elucidated the effect of romosozumab on bone regeneration was not investigated in the current study. A previous in vitro study demonstrated that sclerotin antibody normalized mineralization defect induced by exogenous sclerostin in osteoblast lineage cell-based mineralization assay [24]. An additional study in mouse showed that sclerostin antibody converted bone-lining cells into active osteoblasts [25]. Clinical study demonstrated that romosozumab primarily increased modeling-based bone formation [26]. Increased modeling-based bone formation can play an important role in the process of bone regeneration. Mechanism of action of romosozumab on bone regeneration remains an important area for future research.

Other osteoporosis agents have not been evaluated in the same model of nonhuman primates presented here. To date, only PTH 1–34 and romosozumab were reported in osteotomy models in nonhuman primates. PTH improved callus maturity but not ultimate load and stiffness in healing femurs after 26 weeks [27]. Romosozumab treatment increased callus maturity and strength in healing fibulae after 10 weeks [28]. Numerical improvements in callus bone density and stiffness were also evident in romosozumab-treated osteotomized ulna after 16 weeks in a separate study [29]. However, in randomized controlled clinical studies, clinical doses of teriparatide and romosozumab did not accelerate radiographic healing or improve function as the primary endpoints [[11], [12], [13], [14]]. The reasons for the lack of clinical translation are unknown and may be related to the differences in the methodologies evaluating the healing response, surgical fixation, the dose used or treatment duration between clinical and preclinical studies. Clinical trials usually evaluate radiographic healing or functional assessments in contrast to preclinical studies that focus on biomechanical strength or callus maturity at the fracture site [30]. This discrepancy highlights the need to use clinical endpoints such as radiographic healing in a preclinical model. The study presented here is intended to address this need.

One of the barriers for patients with fragility fracture receiving osteoporosis treatment is the perceived negative effects of anti-resorptive agents on the healing process. Romosozumab is a bone-forming agent with the dual action of increasing bone formation and decreasing bone resorption. In phase 2 clinical studies of fracture healing, no delayed healing or nonunion was observed in romosozumab-treated patients [13,14]. In addition, in the post hoc analyses of the phase 3 FRAME and ARCH clinical studies, delayed healing and nonunion were not observed in romosozumab-treated postmenopausal women with osteoporosis [31]. These results support the use of romosozumab immediately after osteoporotic fracture to increase bone strength and reduce the subsequent increase in fracture risk that occurs after a fracture [31,32].

Partial bone regeneration response in the vehicle group (ie, DBM alone) in this model is encouraging, and at first glance, it may appear that DBM can partially promote bone repair. Previous studies in large animals including nonhuman primates demonstrated osteoinductive activity of DBM [33,34]. However, in this study, we did not include a control group without DBM. Therefore, the current study does not provide conclusive evidence of the osteoinductive effect of DBM in this model. Osteoinductive activity of DBM has not been demonstrated in humans. Another interesting visual observation from the micro-CT images of the bone defect is that the cortical bone appeared to be less dense in vehicle-treated monkeys (Fig. S4). Previous studies have demonstrated that sclerostin mediates bone response to mechanical unloading in mice [35]. Sclerostin inhibition increased bone formation and bone mass when given to prevent or restore bone loss in several animal models of immobilization. including a model of spinal cord injury [[36], [37], [38], [39], [40]]. Recently, a small single-arm clinical study (n = 12) demonstrated that romosozumab significantly increased BMD at the lumbar spine and total hip in women with spinal cord injury [41].

There are some limitations in this study. First, the dose of romosozumab used in this study was tenfold multiple of the clinically approved dose for osteoporosis treatment in humans [17].Thus, it is impossible to predict if the approved dose for osteoporosis in humans will be sufficient to translate these findings in critical-size defect in humans. Nevertheless, a couple of case reports demonstrated encouraging findings with clinical dose of romosozumab in other conditions [22,23]. Although this dose of romosozumab (30 mg/kg, twice monthly) has not been evaluated in humans, another sclerostin antibody was administered at 20 mg/kg or 40 mg/kg monthly up to 6 months in patients with osteogenesis imperfecta and appeared to be safe in subjects with osteogenesis imperfecta [42]. Importantly, the high dose of romosozumab used in monkeys appeared to be well tolerated, with no adverse impact on bone regeneration. Second, this study used DBM alone, not in combination with a bone graft, which is usually the case in the clinical setting. Thus, it remains unknown how including a bone graft along with DBM would have impacted the bone regeneration response. Third, the study did not evaluate the combination of romosozumab with other bone graft substitutes or 3D printing materials. 3D bioprinting appears to be a promising technology for bone regeneration [43]. Fourth, the bone metabolic rate of nonhuman primates differs from that of humans. Further studies are required to understand whether the observed bone regeneration promoting effect of romosozumab can be replicated in humans or not.

In conclusion, this study demonstrated that high-dose romosozumab in combination with DBM improved bone regeneration in a critical-size bone defect model and increased bone mass in nonsurgical bone in nonhuman primates. A prior pre-clinical study in rodents also demonstrated that romosozumab may be effective in enhancing bone formation in the presence of a bone defect when combined with rhBMP2 [21]. These results support the concept of evaluating romosozumab in combination with a bone graft or an osteoinductive bone graft substitute in a challenging-to-heal condition. Further studies are required to define the clinical utility of romosozumab in improving bone regeneration in humans.

## CRediT authorship contribution statement

**Xiaodong Li:** Conceptualization, Investigation, Methodology, Supervision, Visualization, Writing – original draft, Writing – review & editing. **Frank Asuncion:** Data curation, Formal analysis, Writing – review & editing. **Michael Ominsky:** Data curation, Formal analysis, Visualization, Writing – review & editing. **Qing-Tian Niu:** Data curation, Formal analysis, Writing – review & editing. **Kristina E. Akesson:** Writing – review & editing. **Jeffrey Wang:** Writing – review & editing. **Jay Lieberman:** Writing – review & editing. **Hua Zhu Ke:** Writing – review & editing, All authors have read and approved the final submitted manuscript.

## Ethics statement

Animal study was approved by the WMB Institutional Animal Care and Use Committee (WMA201201), and the study was conducted according to the study protocol and current Guangxi Weimei Bio-tech Co. guidance documents and standard operating procedures.

## Funding

10.13039/100002429Amgen Inc. and UCB sponsored this study.

## Declaration of competing interest

Xiaodong Li: (employee and stock owner: Amgen Inc.); Frank Asuncion, Michael Ominsky, Qing-Tian Niu, Hua Zhu Ke (former employees and stock owners: Amgen Inc.); Kristina E Akesson (lecture fees: Amgen Inc., Astellas Pharma, UCB); Jeffrey Wang (royalties: Biomet, Seaspine, Synthes, Novapproach, GS Medical; investments/options: Bone Biologics, Pearldiver, Electrocore, Surgitech, Illuminant; consulting: Bioretec, Angitia, Epidutech, Depuy, Moving Spine; board of directors: AO Foundation, National Spine Health Foundation; editorial boards: Global Spine Journal Editor-in-Chief; fellowship funding (paid to institution): AO Foundation) and Jay Lieberman (none).

## References

[1] Xue N., Ding X., Huang R., Jiang R., Huang H., Pan X. (2022). Bone tissue engineering in the treatment of bone defects. Pharmaceuticals.

[2] Roddy E., DeBaun M.R., Daoud-Gray A., Yang Y.P., Gardner M.J. (2018). Treatment of critical-sized bone defects: clinical and tissue engineering perspectives. Eur J Orthop Surg Traumatol.

[3] Virk M.S., Lieberman J.R. (2012). Biologic adjuvants for fracture healing. Arthritis Res Ther.

[4] Alaee F., Virk M.S., Tang H., Sugiyama O., Adams D.J., Stolina M. (2014). Evaluation of the effects of systemic treatment with a sclerostin neutralizing antibody on bone repair in a rat femoral defect model. J Orthop Res.

[5] Virk M.S., Alaee F., Tang H., Ominsky M.S., Ke H.Z., Lieberman J.R. (2013). Systemic administration of sclerostin antibody enhances bone repair in a critical-sized femoral defect in a rat model. J Bone Joint Surg Am.

[6] Padhi D., Jang G., Stouch B., Fang L., Posvar E. (2011). Single-dose, placebo-controlled, randomized study of AMG 785, a sclerostin monoclonal antibody. J Bone Miner Res.

[7] McClung M.R., Grauer A., Boonen S., Bolognese M.A., Brown J.P., Diez-Perez A. (2014). Romosozumab in postmenopausal women with low bone mineral density. N Engl J Med.

[8] Cosman F., Crittenden D.B., Adachi J.D., Binkley N., Czerwinski E., Ferrari S. (2016). Romosozumab treatment in postmenopausal women with osteoporosis. N Engl J Med.

[9] Saag K.G., Petersen J., Brandi M.L., Karaplis A.C., Lorentzon M., Thomas T. (2017). Romosozumab or alendronate for fracture prevention in women with osteoporosis. N Engl J Med.

[10] Langdahl B.L., Libanati C., Crittenden D.B., Bolognese M.A., Brown J.P., Daizadeh N.S. (2017). Romosozumab (sclerostin monoclonal antibody) versus teriparatide in postmenopausal women with osteoporosis transitioning from oral bisphosphonate therapy: a randomised, open-label, phase 3 trial. Lancet.

[11] Bhandari M., Jin L., See K., Burge R., Gilchrist N., Witvrouw R. (2016). Does teriparatide improve femoral neck fracture healing: results from a randomized placebo-controlled trial. Clin Orthop Relat Res.

[12] Aspenberg P., Malouf J., Tarantino U., García-Hernández P.A., Corradini C., Overgaard S. (2016). Effects of teriparatide compared with risedronate on recovery after pertrochanteric hip fracture: results of a randomized, active-controlled, double-blind clinical trial at 26 weeks. J Bone Joint Surg Am.

[13] Bhandari M., Schemitsch E.H., Karachalios T., Sancheti P., Poolman R.W., Caminis J. (2020). Romosozumab in skeletally mature adults with a fresh unilateral tibial diaphyseal fracture: a randomized phase-2 study. J Bone Joint Surg Am.

[14] Schemitsch E.H., Miclau T., Karachalios T., Nowak L.L., Sancheti P., Poolman R.W. (2020). A randomized, placebo-controlled study of romosozumab for the treatment of hip fractures. J Bone Joint Surg Am.

[15] Khan S.N., Fraser J.F., Sandhu H.S., Cammisa F.P., Girardi F.P., Lane J.M. (2005). Use of osteopromotive growth factors, demineralized bone matrix, and ceramics to enhance spinal fusion. J Am Acad Orthop Surg.

[16] Ominsky M.S., Niu Q.-T., Li C., Li X., Ke H.Z. (2014). Tissue-level mechanisms responsible for the increase in bone formation and bone volume by sclerostin antibody. J Bone Miner Res.

[17] Ominsky M.S., Boyd S.K., Varela A., Jolette J., Felx M., Doyle N. (2017). Romosozumab improves bone mass and strength while maintaining bone quality in ovariectomized cynomolgus monkeys. J Bone Miner Res.

[18] Turk J.R., Deaton A.M., Yin J., Stolina M., Felx M., Boyd G. (2020). Nonclinical cardiovascular safety evaluation of romosozumab, an inhibitor of sclerostin for the treatment of osteoporosis in postmenopausal women at high risk of fracture. Regul Toxicol Pharmacol.

[19] Boyce R.W., Niu Q.-T., Ominsky M.S. (2017). Kinetic reconstruction reveals time-dependent effects of romosozumab on bone formation and osteoblast function in vertebral cancellous and cortical bone in cynomolgus monkeys. Bone.

[20] Marsell R., Einhorn T.A. (2011). The biology of fracture healing. Injury.

[21] Tinsley B.A., Dukas A., Pensak M.J., Adams D.J., Tang A.H., Ominsky M.S. (2015). Systemic administration of sclerostin antibody enhances bone morphogenetic protein-induced femoral defect repair in a rat model. J Bone Joint Surg Am.

[22] Lee S.Y., Kawasaki K., Inagaki K. (2022). Successful treatment of humeral shaft nonunion with romosozumab: a case report. Trauma Case Rep.

[23] Uemura T., Yano K., Takamatsu K., Miyashima Y., Yasuda H., Konishi S. (2021). Bone healing of distal radius nonunion treated with bridge plating with bone graft substitutes in combination with systemic romosozumab administration: a case report. Jt Dis Relat Surg.

[24] Li X., Ominsky M.S., Warmington K.S., Morony S., Gong J., Cao J. (2009). Sclerostin antibody treatment increases bone formation, bone mass, and bone strength in a rat model of postmenopausal osteoporosis. J Bone Miner Res.

[25] Kim S.W., Lu Y., Williams E.A., Lai F., Lee J.Y., Enishi T. (2017). Sclerostin antibody administration converts bone lining cells into active osteoblasts. J Bone Miner Res.

[26] Eriksen E.F., Chapurlat R., Boyce R.W., Shi Y., Brown J.P., Horlait S. (2022). Modeling-based bone formation after 2 months of romosozumab treatment: results from the FRAME clinical trial. J Bone Miner Res.

[27] Manabe T., Mori S., Mashiba T., Kaji Y., Iwata K., Komatsubara S. (2007). Human parathyroid hormone (1-34) accelerates natural fracture healing process in the femoral osteotomy model of cynomolgus monkeys. Bone.

[28] Ominsky M.S., Li C., Li X., Tan H.L., Lee E., Barrero M. (2011). Inhibition of sclerostin by monoclonal antibody enhances bone healing and improves bone density and strength of nonfractured bones. J Bone Miner Res.

[29] Florio M., Kostenuik P.J., Stolina M., Asuncion F.J., Grisanti M., Ke H.Z. (2023). Dual inhibition of the Wnt inhibitors DKK1 and sclerostin promotes fracture healing and increases the density and strength of uninjured bone: an experimental study in nonhuman primates. J Bone Joint Surg Am.

[30] Lopas L.A., Shen H., Zhang N., Jang Y., Tawfik V.L., Goodman S.B. (2023). Clinical assessments of fracture healing and basic science correlates: is there room for convergence?. Curr Osteoporos Rep.

[31] Lane J, Langdahl B, Stone M, Kurth A, Oates M, Timoshanko J, et al. Romosozumab in patients who experienced an on-study fracture: post hoc analyses of the FRAME and ARCH phase 3 trials. Osteoporos Int;35(7):1195-1204. 10.1007/s00198-024-07049-w PMC11211143 38573517

[32] Lindsay R., Silverman S.L., Cooper C., Hanley D.A., Barton I., Broy S.B. (2001). Risk of new vertebral fracture in the year following a fracture. JAMA.

[33] Ozdemir M.T., Kir M. (2011). Repair of long bone defects with demineralized bone matrix and autogenous bone composite. Indian J Orthop.

[34] Louis-Ugbo J., Murakami H., Kim H.S., Minamide A., Boden S.D. (2004). Evidence of osteoinduction by Grafton demineralized bone matrix in nonhuman primate spinal fusion. Spine.

[35] Lin C., Jiang X., Dai Z., Guo X., Weng T., Wang J. (2009). Sclerostin mediates bone response to mechanical unloading through antagonizing Wnt/beta-catenin signaling. J Bone Miner Res.

[36] Tian X., Jee W.S., Li X., Paszty C., Ke H.Z. (2011). Sclerostin antibody increases bone mass by stimulating bone formation and inhibiting bone resorption in a hindlimb-immobilization rat model. Bone.

[37] Qin W., Li X., Peng Y., Harlow L.M., Ren Y., Wu Y. (2015). Sclerostin antibody preserves the morphology and structure of osteocytes and blocks the severe skeletal deterioration after motor-complete spinal cord injury in rats. J Bone Miner Res.

[38] Beggs L.A., Ye F., Ghosh P., Beck D.T., Conover C.F., Balaez A. (2015). Sclerostin inhibition prevents spinal cord injury-induced cancellous bone loss. J Bone Miner Res.

[39] Zhao W., Li X., Peng Y., Qin Y., Pan J., Li J. (2018). Sclerostin antibody reverses the severe sublesional bone loss in rats after chronic spinal cord injury. Calcif Tissue Int.

[40] Zhang D., Hu M., Chu T., Lin L., Wang J., Li X. (2016). Sclerostin antibody prevented progressive bone loss in combined ovariectomized and concurrent functional disuse. Bone.

[41] Edwards W.E., Crack L.E., Muresan T., Simonian N., Schnitzer T.J. (2023). Treatment with monthly romosozumab injections increases areal bone mineral density at the lumbar spine and hip in women with chronic spinal cord injury. J Bone Miner Res.

[42] Gottesman G.S., Carpenter T.O., Wallace M., Smith P., Imel E.A., Wang H. (2024). Assessing the efficacy and safety of setrusumab for osteogenesis imperfecta: updated phase 2 data from the phase 2/3 orbit study. J Endocr Soc.

[43] Li H., Mao B., Zhong J., Li X., Sang H. (2024). Localized delivery of metformin via 3D printed GelMA-Nanoclay hydrogel scaffold for enhanced treatment of diabetic bone defects. J Orthop Translat.

