# Supplementary material for: High-dose romosozumab promoted bone regeneration of critical-size ulnar defect filled with demineralized bone matrix in nonhuman primates
Source: J Orthop Translat. 2025 Jul 10;54:1–7. doi: 10.1016/j.jot.2025.06.019 (PMC12343352; doi:10.1016/j.jot.2025.06.019)
Supplement: Multimedia component 1 [file mmc1.docx]

**Romosozumab Promoted Bone Regeneration of Critical-Size Ulnar Defect Filled with Demineralized Bone Matrix in Nonhuman Primates**

**SUPPLEMENTARY MATERIAL**

**Fig. S1: In vivo x-ray images of ulnae with best bone regeneration responses in vehicle- and romosozumab-treated cynomolgus monkeys at different time points**

**
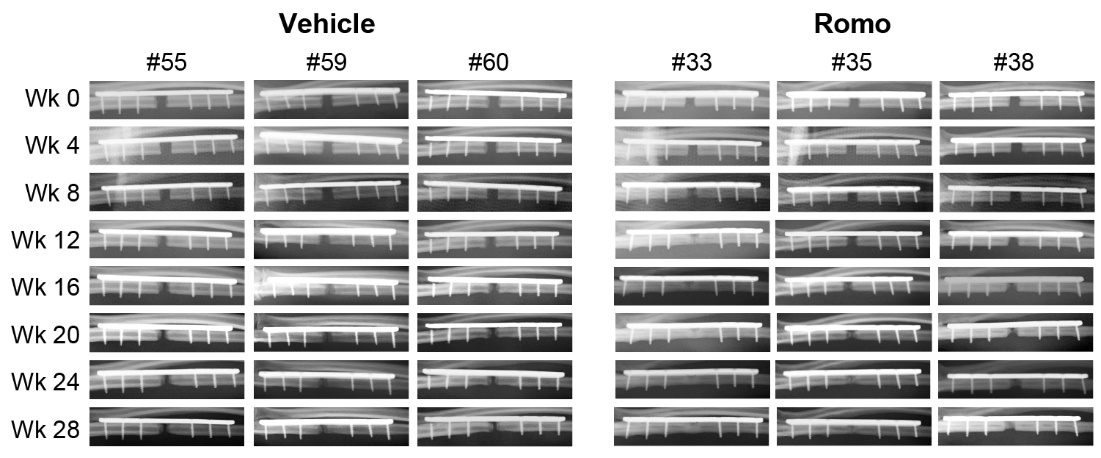
**

X-ray images of the three best responders from monkeys treated with vehicle and romosozumab at different time points after drug administration. # = number represents the individual monkey number. Romo, romosozumab.

**Fig. S2: In vivo x-ray images of ulnae of all monkeys at week 28 week with metal plates intact in vehicle- and romosozumab-treated cynomolgus monkeys**

**
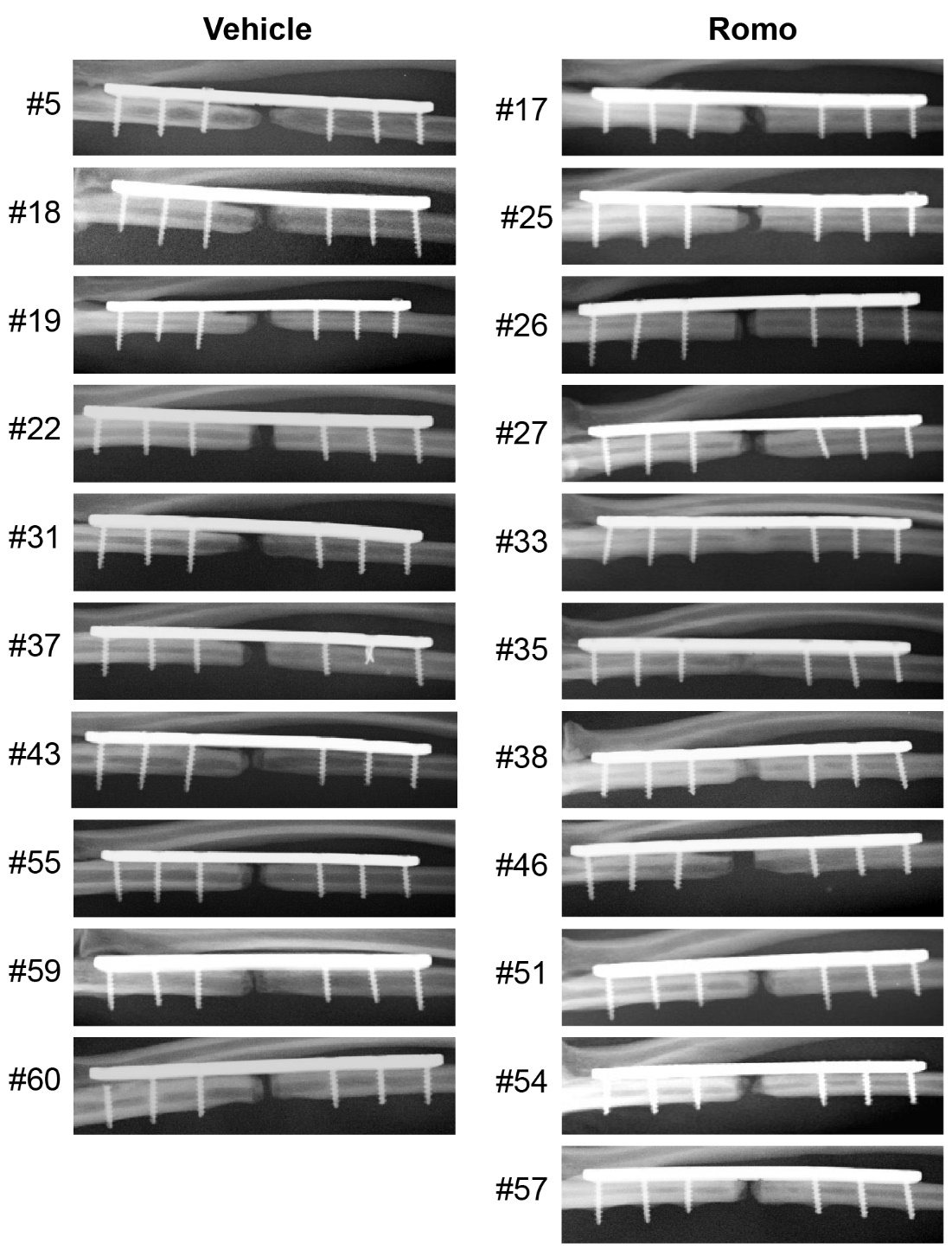
**

One monkey was excluded from the romosozumab group due to undetectable serum romosozumab concentration from week 6 to the end of the study. # = number represents the individual monkey number. Romo, romosozumab.

**Fig. S3: Ex vivo x-ray images of ulnae of all monkeys at week 28 in vehicle- and romosozumab-treated cynomolgus monkeys**

**
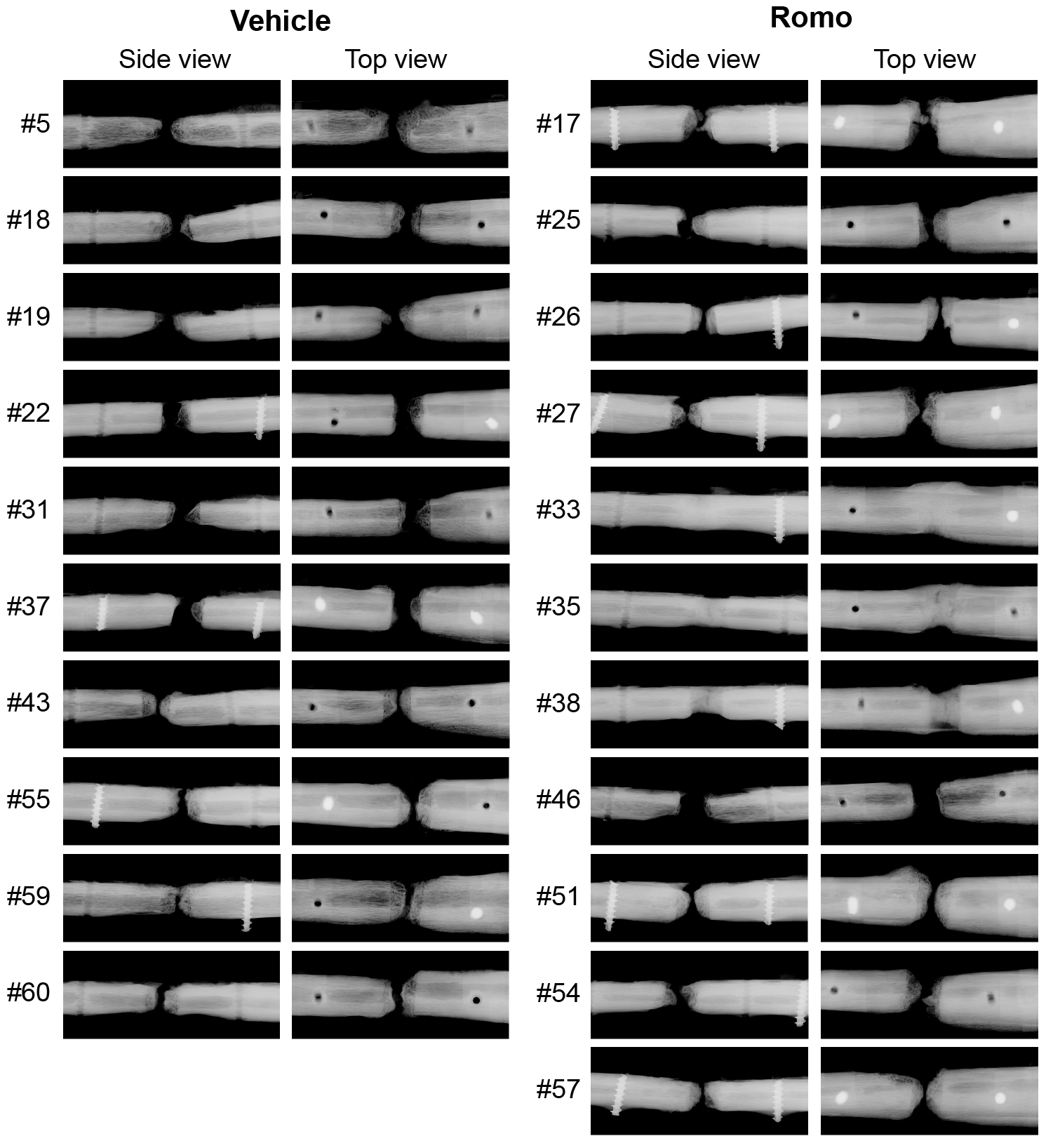
**

One monkey was excluded from the romosozumab group due to undetectable serum romosozumab concentration from week 6 to the end of the study. # = number represents the individual monkey number. Romo, romosozumab.

**Fig. S4: Micro-CT images of ulnae of all cynomolgus monkeys at 28 weeks in vehicle- and romosozumab-treated group**


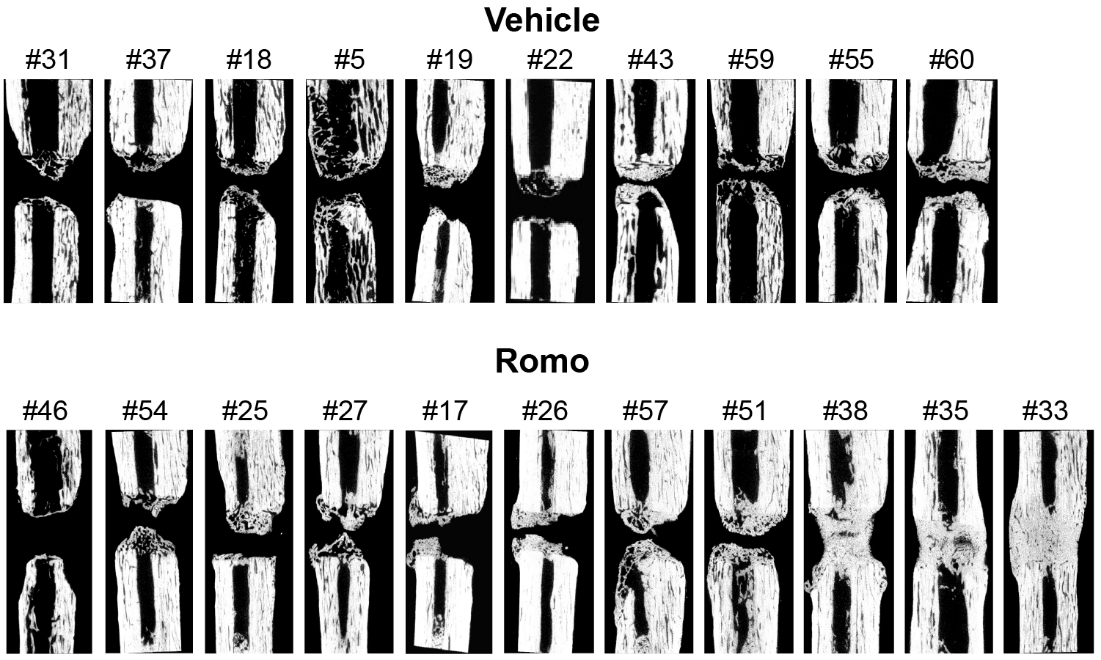


Representative micro-CT images on a 2D sagittal plane along the ulnar diaphysis. Images are ranked by values of the new bone area within the defect region from low (left) to high (right). The last three images in each group are the best three responders (corresponding to images shown in Fig. 3). One monkey was excluded from the romosozumab group due to undetectable serum romosozumab concentration from week 6 to the end of the study. # = number above images represents the individual monkey number. Micro-CT, micro-computed tomography; Romo, romosozumab.
