# Supplementary material for: High-dose romosozumab promoted bone regeneration of critical-size ulnar defect filled with demineralized bone matrix in nonhuman primates
Source: J Orthop Translat. 2025 Jul 10;54:1–7. doi: 10.1016/j.jot.2025.06.019 (PMC12343352; doi:10.1016/j.jot.2025.06.019)
Supplement: Multimedia component 2 [file mmc2.zip › Cynos Ulnar healing_JOT COI_Signed_02.24.2025.rtf]

Journal of Orthopaedic Translation


AUTHORSHIP & CONFLICTS OF INTEREST STATEMENT
 
 
Manuscript title: High-Dose Romosozumab Promoted Bone Regeneration of Critical-Size Ulnar Defect Filled with Demineralized Bone Matrix in Nonhuman Primates 

AUTHORSHIP 
All persons who meet authorship criteria are listed as authors, and all authors certify that they have participated sufficiently in the work to take public responsibility for the content, including participation in the concept, design, analysis, writing, or revision of the manuscript. Each author certifies that this material or part thereof has not been published in another journal, that it is not currently submitted elsewhere, and that it will not be submitted elsewhere until a final decision regarding publication of the manuscript in Journal of Orthopaedic Translation has been made. 
Indicate the specific contributions made by each author (list the authors' initials followed by their surnames, e.g., Y.L. Cheung). The name of each author must appear at least once in each of the three categories below. 

Category 1 
Conception and design of study: Xiaodong Li

acquisition of data: Frank Asuncion, Michael Ominsky, Qing-Tian Niu

analysis and/or interpretation of data: Frank Asuncion, Michael Ominsky, Qing-Tian Niu

Category 2 
Drafting the manuscript: Xiaodong Li

revising the manuscript critically for important intellectual content: Xiaodong Li, Frank Asuncion, Michael Ominsky, Qing-Tian Niu, Kristina E Akesson, Jeffrey Wang, Jay Lieberman, Hua Zhu Ke

Category 3 
Approval of the version of the manuscript to be published (the names of all authors must be listed): Xiaodong Li, Frank Asuncion, Michael Ominsky, Qing-Tian Niu, Kristina E Akesson, Jeffrey Wang, Jay Lieberman, Hua Zhu Ke

Acknowledgements 
All persons who have made substantial contributions to the work reported in the manuscript (e.g., technical help, writing and editing assistance, general support), but who do not meet the criteria for authorship, are named in the Acknowledgements and have given us their written permission to be named. If we have not included an Acknowledgements, then that indicates that we have not received substantial contributions from non-authors. 
 
This study was funded by Amgen Inc. and UCB. The authors would like to thank Guangxi Weimei Bio-Tech Co for conducting the study; Dr. Rogely Boyce for her advice on fluorochrome label schedule; Dr. Hossein Salimi-Moosavi, Dr. Theingi Thway, and the research team for measurement of serum romosozumab; Mario Grisanti and Hong Lin Tan for their technical support. Medical writing support was provided by Lisa Humphries, PhD, of Amgen Inc. and Varsha Jain, PhD, of Cactus Life Sciences (part of Cactus Communications) and funded by Amgen Inc.
Article Processing Charge 
 The corresponding author agrees to pay the Journal of Orthopaedic Translation Article Processing Charge upon acceptance of the work for publication in Journal of Orthopaedic Translation, unless prior arrangements have been made to waive the Article Processing Charge. 
 
 
		
_____Xiaodong Li_________ 　@　@ _____Xiaodong Li__________________  　@      01.20.2025
Corresponding Author Name 　@　@　@ Corresponding Author Signature  　@     Date 
(printed) 
 
 
CONFLICTS OF INTEREST 
A conflict of interest occurs when an individual's objectivity is potentially compromised by a desire for financial gain, prominence, professional advancement or a successful outcome. The Editors of the Journal of Orthopaedic Translation strive to ensure that what is published in the Journal is as balanced, objective and evidence-based as possible. Since it can be difficult to distinguish between an actual conflict of interest and a perceived conflict of interest, the Journal requires authors to disclose all and any potential conflicts of interest. 

Section I 
The authors whose names are listed immediately below certify that they have NO affiliations with or involvement in any organization or entity with any financial interest (such as honoraria; educational grants; participation in speakers' bureaus; membership, employment, consultancies, stock ownership, or other equity interest; and expert testimony or patent-licensing arrangements), or non-financial interest (such as personal or professional relationships, affiliations, knowledge or beliefs) in the subject matter or materials discussed in this manuscript. 

Author names: 

Jay Lieberman


Section II 
The authors whose names are listed immediately below report the following details of affiliation or involvement 
in an organization or entity with a financial or non-financial interest in the subject matter or materials 
discussed in this manuscript. Please specify the nature of the conflict on a separate sheet of paper if the space 
below is inadequate. 

Author names and details of the conflict(s) of interest: 

Xiaodong Li: employee and stock owner: Amgen Inc.
Frank Asuncion, Michael Ominsky, Qing-Tian Niu, Hua Zhu Ke: former employees and stock owners: Amgen Inc.
Kristina E. Akesson: lecture fees: Amgen Inc., Astellas Pharma, UCB
Jeffrey Wang: royalties: Biomet, Seaspine, Synthes, Novapproach, GS Medical; investments/options: Bone Biologics, Pearldiver, Electrocore, Surgitech, Illuminant; consulting: Bioretec, Angitia, Epidutech, Depuy, Moving Spine; board of directors: AO Foundation, National Spine Health Foundation; editorial boards: Global Spine Journal Editor-in-Chief; fellowship funding (paid to institution): AO foundation

This Authorship & Conflicts of Interest Statement is signed by all the authors listed in the manuscript to  indicate agreement that the above information is true and correct (a photocopy of this form may be used if there are more than 10 authors):
 
Author's name (typed)  　@　@　@　@	Author's signature    　@　@　@　@	Date

Xiaodong Li
_______________________   	____ Xiaodong Li ____________ 	  _1/25/2025_____________

Frank Asuncion
_______________________   	_______________________ 	____02/13/2025______
Michael Ominsky_______    _____________   ____02/14/2025__________


Qing-Tian Niu
_______________________   _______ Qingtian Niu ________________       2/13/2025_______________
	
Kristina E Akesson                           1/30/2025___________               	        ____________


Jeffrey Wang
_______________________   	____ Jeffrey Wang _________   	_______1/29/2025_________

Jay Lieberman                        
________________                                                      ______02/19/25_________

Hua Zhu Ke01/25
_______________________   	  _______________________     _____02/05/2025___________


_______________________ 		_______________________  	_______________________


_______________________  	_______________________  	_______________________
	
